# 

**Published:** 1884-01-20

**Authors:** 


### TABLE OF CONTENTS.

ORIGINAL AND SELECTED ARTICLES.

Ligation of Primitive Carotid Artery for Traumatic Aneurism, by Jno. Thad. Johnson, M.D., Professor of Surgery Southern Medical College, Atlanta, Ga., 1. Clinical Remarks on Dropsy, by G. G. Roy, M.D, Professor of Materia Medica and Lecturer on Toxicology in Southern Medical College, Atlanta, Ga., 5. The Case of John Jolly, by A. W. Reese, M.D., of Missouri, 8. Ingrowing Toe-Nail, by R. C. Word, M.D., Professor of Physiology in Southern Medical College, Atlanta, Ga., 13. The Treatment of Piles, by William H. Veatch, M.D, of Carthage, Ill., 14. Cold, Hot and Warm Applications in the Treatment of Diseases of the Eye, by J. Morrison Ray, M.D., 17. A Toxic Dose of Belladonna, by H. Gibbons Sr., M.D., 20. Sierra Salviæ in Remittent Fever, by Francis A. Evans, M.D., Newbern, Tenn., 22. The Ether Douche or Lavement for Local Pain, by C. H. Hughes, M.D., 23. Iodide of Potassium in the Treatment of Typhoid Fever, 24.

ABSTRACTS AND GLEANINGS.

Maximum of Carbolic Acid in Antiseptic Gauze; Insomnia, 25. Dropsy; Bowditch's Formula for Irregular Heart, 26. A New Contrivance for Raising Patients; Treatment of Premature Baldness; Micrococci. 27. Treatment of Gonorrhœa; Iodoform in Ophthalmic Practice; Wm. Harvey's Remains, 28 Painting with Tincture of Iodine in Variola; Injections into the Optic Nerve; Preserving Cadavers; Constipation; Snake-Bite, 29. Eucalyptus in the Treatment of Acute Tonsillitis; Salicylic Acid for Venereal Warts and Ulcers; Opium Poisoning, 30.

SCIENTIFIC ITEMS.

Sugar from Rags; Terra Cotta Lumber; Styrian Steel, 31. Photographic Progress; New Electric Power; A Plastic Mass, 32.

PRACTICAL NOTES AND FORMULÆ.

Sweating in Phthisis; External Application in Acute Rosacea; Croton Chloral in Whooping Cough; Lumbago, 33 Acute Tonsillitis; Laville's Gout Mixture; Injections for Gonorrhœa, 34. Eczema of the Scalp in Infants; Depilatories; Dandruff; Brandreth's Pills, 35. Paste or Glue for Paper Labels; For Syphilis; Tonic Bitters; Chronic Rheumatism and Neuralgia; Cure for Snake-Bites; Chilblains, 36.

EDITORIALS AND MISCELLANEOUS.

The New Year; Retrospect of Medical Progress, 37. Friendship of Doctors; Too Many Free Samples; Beautiful Samples; Lambert & Co,; Imperial Granum, 38. Book Notices; Pamphlets and Books Received, 39. Receipted; Special Notices, 40.

